# Parental Home Monitoring and Support and Students’ Online Learning and Socioemotional Well-Being During COVID-19 School Suspension in Hong Kong

**DOI:** 10.3389/fpsyg.2022.916338

**Published:** 2022-06-02

**Authors:** Cheng Yong Tan, Qianqian Pan, Yuxiao Zhang, Min Lan, Nancy Law

**Affiliations:** ^1^Faculty of Education, The University of Hong Kong, Hong Kong, Hong Kong SAR, China; ^2^National Institute of Education, Nanyang Technological University, Singapore, Singapore; ^3^Department of Educational Studies, College of Education, Purdue University, West Lafayette, IN, United States; ^4^College of Teacher Education, Zhejiang Normal University, Jinhua, China

**Keywords:** COVID-19, online learning, parental involvement, school closure, self-efficacy, self-regulation, socioeconomic status, socioemotional learning

## Abstract

Contextualized in the prolonged period of COVID-19-related school suspension in Hong Kong, the present study unravels relationships among socioeconomic status (SES), parental involvement, and learning outcomes for a matched sample of 186 primary and 932 secondary school students and their parents who participated in the eCitizen Education 360 survey. Three-step latent profile analysis (LPA) revealed different types of parental involvement at home and in school. For the primary school sample, students’ SES did not predict membership in the parental involvement typology, but students whose parents provided more home monitoring and support had the highest level of online self-efficacy. As for the secondary student sample, students whose parents provided more home monitoring and support tended to have access to more home learning resources. Students whose parents provided home monitoring and support had the highest levels of online self-efficacy, acquisition of digital skills, and cognitive-emotional regulation, and were the least worried about school resumption. The study underscores complex patterns of parental involvement and identifies effective parental involvement practices that contribute to students’ home online learning during the school suspension.

## Introduction

Students in many education systems experienced prolonged periods of school suspension during COVID-19. For example, students in Hong Kong switched from face-to-face lessons in school to home-based online learning in January–May/June 2020 as the city implemented various measures to contain the community spread of the virus. The transition in learning modes affected student learning in many ways. First, compared to face-to-face school-based lessons, the effectiveness of home online learning is influenced by the availability of socioeconomic status (SES)-related family resources and parental involvement (Livingstone et al., 2018; Bol, 2020). Second, students need to be self-regulated and self-efficacious to manage their own learning during online sessions (Hwang et al., 2017; Eyimaya and Irmak, 2021). Third, students struggle to cope with the uncertainty of learning modality during and after the school suspension (Spinelli et al., 2020).

Given the different contexts in which students learn during the school suspension period as compared to pre-pandemic times, it is imperative to advance our understanding of how SES, parental involvement, students’ socioemotional well-being, and online learning are related in three key areas. First, we need to unravel what parents are doing to support students’ learning and well-being, especially in lower-SES families during home-based online learning (Kerr et al., 2021; Low and Mounts, 2021; Radey et al., 2021; Fosco et al., 2022; Ilari et al., 2022). Studies have found that parents encounter different challenges in trying to support student learning during the school suspension (Agaton and Cueto, 2021; Akinrinmade et al., 2021; Alshwiah, 2021; Chen C. Y. C. et al., 2022), so insights gleaned from research in this area will advance our knowledge on social stratification and equity in student learning.

Second, we need to understand how parental involvement contributes to students’ learning and socioemotional well-being during the school suspension (Demir and Demir, 2021; Lawrence and Fakuade, 2021; Liu et al., 2021; Yang et al., 2022). The knowledge gleaned from research in this area can inform parental involvement practices in the *New Normal* of education—situations that call for agile switching across blended, fully online (asynchronous or bichronous), or hybrid (combining face-to-face and online synchronous at the same time) learning modes (Martin et al., 2020; Morse et al., 2022), which may be compelled by future episodes of mandatory school suspension or in support of pedagogical innovation initiatives.

Third, the onset of sustained home-based online learning during school suspension underscores the importance of and challenges to ensuring students’ socioemotional well-being (Mactavish et al., 2021). Before the pandemic, some schools may have experienced a tension between prioritizing students’ academic achievement and enhancing students’ socioemotional well-being. However, the school suspension has challenged this bifurcation because students need to have high levels of socioemotional well-being to be adaptive during sustained home-based online learning without face-to-face social support from friends and teachers (Durna and Kosterelioglu, 2021; Allen et al., 2022). The knowledge gleaned from research on students’ socioemotional well-being during the school suspension can inform the design of school-based support programs that enhance students’ self-efficacy, cognitive-emotional self-regulation, and ability to cope with anxiety (Anderson et al., 2021; Hatzichristou et al., 2021; Lorenzo et al., 2021; Wang et al., 2021).

The present study addresses these three key areas by investigating how a specific aspect of family factors, namely parental involvement, can contribute to primary and secondary students’ online learning during the school suspension in Hong Kong.

### Parental Involvement

The study builds on the conceptualization of parental involvement as comprising home and school involvement. For example, Epstein (1995) conceptualized parental involvement as creating a conducive home environment for children, communicating with schools, volunteering in school activities, supporting children’s home learning, participating in school decision-making, and collaborating with the community. Lareau (2003) examined how parents communicate with their children, negotiate with school authorities for special accommodations to address their children’s learning needs, and organize their children’s time to develop the latter’s potential. These different aspects of parental involvement can be summarized as home and school involvement (Pomerantz et al., 2007). Home involvement includes parent–child discussions, parental monitoring of children’s learning, and parents building a positive relationship with their children (McNeal, 1999). School involvement includes parent–teacher discussions on children’s learning and parental participation in school activities (Castro et al., 2015).

It is noteworthy that the literature on parental involvement is largely contextualized in pre-COVID-19 times when formal student learning occurs in school settings *via* face-to-face lessons, and parents assume supporting roles to complement what teachers do at school. During the pandemic-induced school suspension, students spend large amounts of time having online lessons at home and parents have to assume a wider range of roles such as being the student’s learning supervisor, tutor, and home-schooling teacher (Agaton and Cueto, 2021). Morse et al. (2022) found that Australian caregivers who had home-schooled at least one child during COVID-19 were confronted with challenges such as connecting with the children, managing work-life-school balance, and facilitating home-schooling although they were not trained as teachers. Demir and Demir’s (2021) study found that parents of elementary and secondary school students in Turkey had to ensure that their children followed the lessons and motivate their children. In so doing, they became more aware of what their children were learning and appreciated the value of the teachers and the school more. These studies suggest that the distinction between home and school involvement is being blurred and parents may have different patterns of involvement during the pandemic-induced school suspension. However, research has not investigated parental involvement patterns during the school suspension although there are studies unraveling different patterns of parental attitudes toward home learning (Pratama and Firmansyah, 2021). This is the first knowledge gap that the present study will address using latent profile analysis of the patterns of parental home and school involvement before and during the school suspension.

### Socioeconomic Status and Parental Involvement

Socioeconomic status measures individuals’ hierarchical ranking reflecting their access to or control over valued resources necessary for success (Mueller and Parcel, 1981). Research has shown that SES has an enduring influence on students’ physical, cognitive, and socioemotional development (Bradley and Corwyn, 2002). Common SES measures include parents’ educational attainment and occupational status, family income and home resources (Sirin, 2005). Among the four indicators, studies have shown that parental education and home resources (especially educational) are more strongly associated with students’ learning outcomes (Sirin, 2005).

Parents who are more highly educated may be more involved for many reasons. At a personal level, they have higher levels of personal drive and determination (Crane, 1996). From a cultural perspective, these parents have benefited from the education system, so they are more inclined to be involved in activities that enable their children to also succeed academically (Portes and MacLeod, 1996). At home, they are more likely to provide cognitively stimulating and developmentally appropriate learning experiences for their children, engage their children in intellectually enriching conversations, and possess higher educational aspirations for their children (Reay, 1998; Kellaghan, 2001; Park and Holloway, 2013; Hartas, 2015). They are also more equipped to advise their children on school matters and help with their children’s homework. As for the SES indicator of home resources, parents with more of these resources are able to use these resources to support their children’s learning (Stevenson and Stigler, 1992). Therefore, parents with more home resources are more likely to have higher levels of involvement.

In the domain of school involvement, higher-SES parents may be more confident in engaging with their children’s teachers and they are more proactive in soliciting school resources to support their children’s learning (Reay, 1998). In contrast, lower SES parents may be less involved in their children’s education because of barriers they have to surmount (Hornby and Lafaele, 2011; Wang et al., 2016; Malone, 2017). These barriers include time and resource constraints, poor knowledge of their children’s homework, and communication issues with their children (Wang et al., 2016).

Research on the association between SES and parental involvement during the pandemic-induced school suspension where students have to learn at home is tentative and results are mixed at best, thereby pointing to the second knowledge gap. For example, Agaton and Cueto (2021) found that parents from marginalized families living in rural areas in the Philippines were confronted with various challenges that impeded involvement as the learning supervisor, tutor, and home-schooling teacher in their children’s home-based learning during the pandemic. These challenges included financial difficulties during the lockdown and difficulty in accessing and using technology for online learning. Relatedly, Radey et al. (2021) reported that low-income, single mothers in the United States selected home-based care and schooling during the pandemic because of safety or financial constraints. In another study, Chen C. Y. C. et al. (2022) reported that parents from different SES backgrounds in the United States had different concerns that affected their parental involvement. Parents of low- and lower-middle-SES backgrounds encountered more financial and material hardship during the pandemic. In contrast, parents with higher income were stressed over how to structure their home learning environments and plan home educational and physical activities for their children. In a study examining parental-child relationships and interactions, Ilari et al. (2022) did not find differences in different aspects of parenting among parents with different levels of education in the United States during the lockdown. These parenting aspects were related to positive parenting (using praise and compliments for children), use of discipline in parenting, positive relationships (parental modeling and promoting positive interactions with children), positive parental emotional states, parenting self-efficacy, and routine management of children’s daily activities. The present study will address this second knowledge gap by investigating the pattern of parental involvement for parents varying in their education and home resources, as SES indicators, in the latent profile analysis.

### Parental Involvement and Students’ Socioemotional Well-Being

As teaching and learning is conducted online during the pandemic-induced school suspension, it is important to understand students’ online learning outcomes and socioemotional well-being. However, studies have tended to examine students’ socioemotional well-being rather than students’ online learning outcomes. Research shows that students’ socioemotional well-being is often adversely affected during the pandemic-induced school suspension when lessons are conducted online. For example, Chen Y. et al.’s (2022) longitudinal study found that adolescents in Sweden had higher levels of stress and psychosomatic symptoms and lower levels of happiness following the onset of the pandemic. Mactavish et al. (2021) found that students in Ontario reported worse well-being (e.g., worries, happiness) and psychological distress during the pandemic when compared to pre-pandemic times. Durna and Kosterelioglu (2021) reported that teachers in Turkey perceived that students’ socioemotional well-being was adversely affected during the pandemic. Kamaşak et al. (2022) found that students in Turkey were more introverted, irritable, and pessimistic during the pandemic.

There is empirical evidence that parental involvement influences students’ socioemotional well-being during the pandemic-induced school suspension when teaching and learning is conducted online. Allen et al.’s (2022) study of secondary school students in Australia found that when parents focused on identifying and cultivating their children’s positive attributes, students became more adaptive and resilient during the pandemic. Furthermore, students’ positive reappraisal and emotional processing was significantly associated with their stress-related growth, and these psychological factors mediated the effects of strength-based parenting on their growth. Spinelli et al.’s (2021) Italian study showed that more “chaotic” households had higher levels of parenting stress, parents who were more stressed were less involved in their children’s learning, and students whose parents were less involved were less able to regulate their own emotions. Mactavish et al. (2021) reported that student-perceived social support from family and friends was related to less severe post-traumatic stress symptoms and it attenuated the increase in psychological distress in Ontario. Wang et al. (2021) found that adolescents who received more parental social support had higher levels of positive affect and lower levels of negative affect in the United States. Liu et al. (2021) reported that higher levels of parental academic involvement and lower levels of parent–child communication was associated with higher levels of middle school student depression during the pandemic in China. Yang et al.’s (2022) study showed that middle school students in China had higher levels of affective engagement in their learning if they had involved parents and supportive teachers.

The present study examines how parental involvement is related to students’ cognitive-emotional regulation strategies during the school suspension and their worries about school resumption. Cognitive-emotional regulation strategies include students learning to cope with emotional experiences arousing from negative events, such as their perceived worries for future study and life. Medrano et al. (2016) showed that cognitive-emotional regulation can moderate the effects induced by emotional states on adolescents’ academic self-efficacy. A healthy parent–child relationship and parental monitoring are both associated with students’ cognitive-emotional regulation strategies (Chen and Chng, 2016; Hapunda et al., 2019). However, it is unclear how students’ cognitive-emotional regulation behaviors can be influenced or protected by parental involvement (Moilanen et al., 2018), particularly with parents and children spending more time together during the pandemic lockdowns.

### Parental Involvement and Students’ Online Learning

Some researchers have investigated how parental involvement influences students’ online learning outcomes. With the rapid development of online learning, some may perceive a lesser need for parental guidance and intervention. However, research shows that online learning may require parents to play an instructional role in their children’s learning (Borup et al., 2013). For instance, a study in Hong Kong found that some children were unable to complete learning tasks independently during the school suspension due to a lack of learning interests and home environment-related limitations (Lau and Lee, 2021). Furthermore, students may pervasively use electronic devices (e.g., for browsing social media) without parental mediation during the school suspension (Lau and Lee, 2021), thereby resulting in lower levels of online learning. Therefore, it is unsurprising when Lawrence and Fakuade (2021) reported from their study that, compared to the adolescents’ learning participation, parental involvement contributed more to the online learning commitment of adolescent learners in Nigeria during the school suspension.

The present study examines two students’ online learning outcomes during the school suspension, namely students’ online learning self-efficacy and acquisition of digital skills. Online learning self-efficacy refers to students’ beliefs in their capacity for online learning. It is positively related to students’ learning outcomes, including satisfaction of online learning, the intention of continuing online learning, and knowledge acquisition (Bates and Khasawneh, 2007; Hodges, 2008; Shen et al., 2013; Alqurashi, 2016). In light of the implementation of home-based online learning due to the pandemic-induced school suspension, students’ learning has become more dependent on their families (Gupta and Jawanda, 2020; Middleton, 2020). Indeed, some studies show that parents’ engagement and high expectations positively impact students’ online learning self-efficacy (Vekiri and Chronaki, 2008; Hsiao et al., 2012). However, these studies were conducted before COVID-19 when parents had the discretion to decide whether to enroll their children in online learning programs and they can adequately prepare themselves to support their children’s online learning (Beck et al., 2014; Curtis and Werth, 2015). In the case of mandatory online learning during the pandemic-induced school suspension, many parents are ill-prepared to assume new responsibilities such as helping their children to stay focused and navigate online learning platforms (ElSaheli-Elhage, 2021; Weaver and Swank, 2021). Therefore, research needs to examine the relationship between parental involvement and students’ online learning outcomes in these different circumstances.

In summary, the present study will address the (third) research gap pertaining to the influence of parental involvement on students’ socioemotional well-being and online learning outcomes. The students’ socioemotional well-being variables comprise their cognitive-emotional regulation during the school suspension and worries for school resumption while the online learning outcomes comprise students’ online learning self-efficacy and acquisition of digital skills.

### Present Study

**FIGURE 1:**
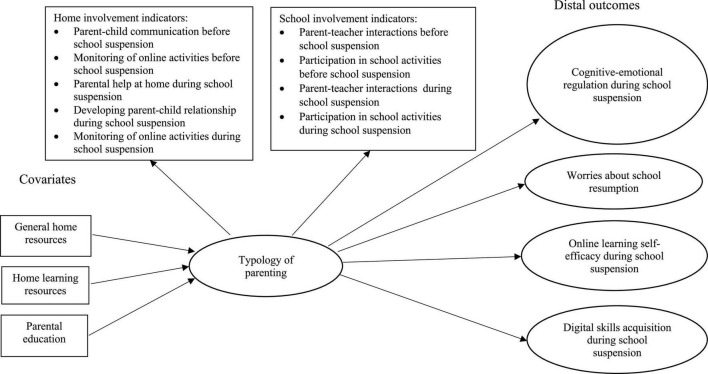
Conceptual framework.

The overall aim of the present study is to examine relationships among SES, parental involvement, and student outcomes during the first wave of school suspension in Hong Kong in 2020. The specific aims are to (a) identify a typology of parental involvement based on variations in their home and school involvement; (b) clarify how SES is associated with different patterns of parental involvement; and (c) compare levels of student outcomes associated with the different patterns of parental involvement using matched data from students and their parents collected in Hong Kong (conceptual framework in Figure 1).

Hong Kong is an interesting context for the present study for two reasons. First, Hong Kong is characterized by high levels of social inequality (Kwan and Wong, 2016), so it is important to ascertain whether there are differences in the pattern of parental involvement among parents of different SES during the pandemic. Second, students in Hong Kong have experienced multiple waves of school suspension since early 2020 as the city implements various measures to contain the community spread of the virus, so it is important to understand what aspects of parental involvement can enhance students’ online learning and mitigate the adverse effects of changing to this learning modality.

The students were sampled from primary and secondary schools. Thus, this study is able to compare the relationships among SES, parental involvement, and students’ outcomes for these two groups of students. This comparison between primary and secondary students is important because students’ developmental needs evolve as they grow up; curricular expectations and educational aims differ with grade levels; and parents may adapt their involvement to address the different learning needs of their children (Hill and Tyson, 2009; Castro et al., 2015; Tan et al., 2019).

## Materials and Methods

### Sample

Matched parent^1^ –child data from primary and secondary schools were analyzed in this study. The sample comprised primary school students and secondary school students as well as their parents who participated in the eCitizen Education 360 survey designed to collect data on students’ learning experiences during school suspension due to COVID-19 in Hong Kong. The primary school sample comprised 186 Primary 3–6 students from 13 primary schools (38.17% from Primary 3–4; 61.83% from Primary 5–6) and their parents. The gender distribution of the primary students was 48.39% boys and 51.61% girls. The primary school students had a mean age of 10.80 years (SD = 1.04; range = [8, 14]). The parents’ educational attainment differed widely (17.20% junior secondary or below; 48.39% senior secondary/diploma; 11.29% associate degree/higher diploma; 19.90% Bachelor’s degree; 6.98% Master’s degree or above). The majority of these parents were aged 36–45 years (64.52%) with the rest aged 19–25 years (0.54%), 26–35 years (9.14%), 46–55 years (23.66%), and 56 years and above (2.15%). The secondary school sample comprised 932 students (66.31% from Secondary 1–3; 33.59% from Secondary 4–6; 33.59%) from 23 secondary schools and their parents. There were more girls (71.46%) than boys (28.43%) in the secondary student sample (0.11% missing data). The secondary school students had a mean age of 14.97 years (SD = 1.58; range = [11, 19]). The parents’ educational attainment of the secondary students was also diverse (21.89% junior secondary or below; 33.91% senior secondary/diploma; 10.09% associate degree/higher diploma; 21.35% Bachelor’s degree; 12.34% Master’s degree or above; 0.43% missing data). The majority of these parents were aged 46–55 years (52.90%) with the rest aged 19–25 years (0.75%), 26–35 years (1.39%), 36–45 years (37.45%), and 56 years and above (7.08%); missing values (0.43%).

### Measures

Three indicators were used to measure SES (adapted from OECD, 2017). The first indicator was computed using student data on whether they had three types of general home resources (desk for study, own room, quiet place to study; *Yes* or *No*). The second indicator measuring the availability of home learning resources was computed using two-parameter logistic model and the graded response model in item response theory for student data on (a) the number of books at home [five-point response scale from *None or very few (0–10 books)* to *Enough to fill three or more bookcases (more than 200 books)*]; and (b) whether they had access to five types of digital learning resources at home (desktop computer, laptop/portable computer, tablet, smartphone, Internet; *Yes* or *No*). The third indicator, parental education, was obtained from parental data on their educational attainment (five-point response scale; *Junior secondary or below* to *Master’s degree or above*).

Parental involvement was measured using parents’ responses to nine questions (adapted from OECD, 2017). Four questions asked about the frequency to which parents were involved in different home involvement practices (four-point scale; from *Never* to *Always*):

• Parent–child communication before the school suspension (three items; e.g., “I discussed with my child what he/she has learnt in school”); and
• Parental monitoring of children’s online activities during the school suspension (three items; e.g., “I controlled my child’s screen time when he/she was using digital devices”); and
• Parental help with homework during the school suspension (three items; e.g., “I helped my child with his/her online homework”);

Parents also responded to another question (four items) on their perceptions of how the pandemic affected the parent–child relationship (e.g., “I have understood my child’s ability more”) using a five-point scale (*Strongly disagree* to *Strongly agree*); items.

Four other questions asked about the frequency with which parents were involved in different school involvement activities (four-point scale; from *Never* to *Always*):

• Parent–teacher interactions before the school suspension (four items; e.g., “I discussed my child’s schoolwork/grades/scores with a teacher on my own initiative”);
• Parental participation in school activities before the school suspension (three items; e.g., “I attended a scheduled teacher-parent meeting”);
• Parent–teacher interactions during the school suspension (four items; e.g., “I discussed my child’s schoolwork/grades/scores with a teacher on my own initiative”); and
• Parental participation in school activities during the school suspension (four items; e.g., “I participated virtually in activities related to parenting organized by the school”).

Separate confirmatory factor analysis (CFA), conducted in the programming environment R using *lavaan* (Rosseel, 2012), for primary and secondary student samples showed that all items significantly loaded on their intended parenting variables, *p* < 0.01 (Tables 1, 2). The CFA model fit (CFI = 1.00, 0.00 ≤ RMSEA ≤ 0.01) and scale reliabilities (0.71 ≤ Ω ≤ 0.95) were satisfactory. Factor scores of the nine parenting variables were used in the analysis.

**TABLE 1:** CFA for parenting variables (primary schools).

Variables	Items	Factor loadings (SE)	CFI	RMSEA	Reliability (Ω)
Parent–child communication before school suspension	par_Q16_11	0.74** (0.08)	1.00	0.00	0.92
	par_Q16_12	0.98** (0.02)			
	par_Q16_13	0.94** (0.02)			
Parental monitoring of children’s online activities before school suspension	par_Q16_8	0.78** (0.06)	1.00	0.00	0.91
	par_Q16_9	1.00** (0.02)			
	par_Q16_10	0.82** (0.04)			
Parental monitoring of children’s online activities during school suspension	par_Q7_7	0.71** (0.06)	1.00	0.00	0.87
	par_Q7_8	1.00** (0.03)			
	par_Q7_9	0.76** (0.05)			
Parental help with homework during school suspension	par_Q7_1	0.64** (0.08)	1.00	0.00	0.71
	par_Q7_2	0.52** (0.09)			
	par_Q7_3	0.84** (0.07)			
Parent–child relationship during school suspension	par_Q11_1	0.81** (0.07)	1.00	0.01	0.81
	par_Q11_2	0.71** (0.09)			
	par_Q11_3	0.70** (0.08)			
	par_Q11_4	0.54** (0.08)			
Parent–teacher interactions before school suspension	par_Q17_1	0.95** (0.02)	1.00	0.00	0.95
	par_Q17_2	0.82** (0.04)			
	par_Q17_3	0.96** (0.01)			
	par_Q17_4	0.82** (0.04)			
Parental participation in school activities before school suspension	par_Q17_5	0.78** (0.10)	1.00	0.00	0.82
	par_Q17_6	0.84** (0.06)			
	par_Q17_7	0.68** (0.07)			
	par_Q17_9	0.74** (0.06)			
Parent–teacher interactions during school suspension	par_Q8_1	0.81** (0.05)	1.00	0.00	0.87
	par_Q8_2	0.54** (0.08)			
	par_Q8_3	0.89** (0.08)			
	par_Q8_4	0.70** (0.06)			
Parental participation in school activities during school suspension	par_Q8_5	0.61** (0.10)	1.00	0.00	0.81
	par_Q8_6	0.84** (0.10)			
	par_Q8_8	0.67** (0.10)			
	par_Q8_10	0.70** (0.09)			

**p < 0.01.

**TABLE 2:** CFA for parenting variables (secondary schools).

Variables	Items	Factor loadings (SE)	CFI	RMSEA	Reliability (Ω)
Parent–child communication before school suspension	par_Q16_11	0.85** (0.02)	1.00	0.00	0.93
	par_Q16_12	0.95** (0.01)			
	par_Q16_13	0.91** (0.02)			
Parental monitoring of children’s online activities before school suspension	par_Q16_8	0.76** (0.02)	1.00	0.00	0.90
	par_Q16_9	0.98** (0.01)			
	par_Q16_10	0.85** (0.02)			
Parental monitoring of children’s online activities during school suspension	par_Q7_7	0.72** (0.02)	1.00	0.00	0.72
	par_Q7_8	0.96** (0.01)			
	par_Q7_9	0.86** (0.02)			
Parental help with homework during school suspension	par_Q7_1	0.67** (0.03)	1.00	0.00	0.89
	par_Q7_2	0.51** (0.04)			
	par_Q7_3	0.83** (0.04)			
Parent–child relationship during school suspension	par_Q11_1	0.81**(0.02)	1.00	0.01	0.84
	par_Q11_2	0.77** (0.03)			
	par_Q11_3	0.78** (0.02)			
	par_Q11_4	0.76** (0.03)			
Parent–teacher interactions before school suspension	par_Q17_1	0.91** (0.01)	1.00	0.00	0.88
	par_Q17_2	0.84** (0.02)			
	par_Q17_3	0.94** (0.01)			
	par_Q17_4	0.79** (0.03)			
Parental participation in school activities before school suspension	par_Q17_5	0.74** (0.04)	1.00	0.00	0.87
	par_Q17_6	0.91** (0.02)			
	par_Q17_7	0.8** (0.02)			
	par_Q17_9	0.75** (0.03)			
Parent–teacher interactions during school suspension	par_Q8_1	0.87** (0.02)	1.00	0.00	0.89
	par_Q8_2	0.78** (0.02)			
	par_Q8_3	0.97** (0.01)			
	par_Q8_4	0.82** (0.02)			
Parental participation in school activities during school suspension	par_Q8_5	0.66** (0.04)	1.00	0.00	0.85
	par_Q8_6	0.79** (0.05)			
	par_Q8_8	0.79** (0.04)			
	par_Q8_10	0.86** (0.03)			

**p < 0.01.

Student outcomes were measured using student responses to four questions on their:

• Cognitive-emotional regulation during the school suspension (three items adapted from Garnefski and Kraaij’s (2007) *Cognitive Emotion Regulation Questionnaire*; e.g., “I thought about how I could best cope with the situation”) using a five-point scale (*Never* to *Always*);
• Worries about school resumption (six items developed by the project team; e.g., “I cannot catch up with my schoolwork”) using a five-point scale (*Strongly disagree* to *Strongly agree*);
• Online learning self-efficacy during the school suspension (six items adapted from Muris’ (2001, 2002) academic self-efficacy subscale; e.g., “I could finish my homework on time”) using a five-point scale (*Strongly disagree* to *Strongly agree*); and
• Acquisition of digital skills during the school suspension (three items developed by the project team; e.g., “Learned new digital skills useful for my academic studies”) using a four-point scale (*Not at all* to *To a large extent*).

Confirmatory factor analysis shows that all items significantly loaded on the intended student outcome variables, *p* < 0.01 (Tables 3, 4). The CFA model fit was satisfactory (0.97 ≤ CFI ≤ 1.00; 0.00 ≤ RMSEA ≤ 0.07). Scale reliabilities (Ω) were 0.56, 0.74, 0.75, and 0.84 for students’ cognitive-emotional regulation, worries about school resumption, online learning self-efficacy, and acquisition of digital skills, respectively. Scale reliabilities were satisfactory for the secondary student sample (0.71 ≤ Ω ≤ 0.82). Factor scores of these student outcome variables were used in the analysis.

**TABLE 3:** CFA for student outcome variables (primary schools).

Variables	Items	Factor loadings (SE)	CFI	RMSEA	Reliability (Ω)
Cognitive-emotional regulation during the school suspension	Q8_1	0.66** (0.06)	1.00	0.00	0.56
	Q8_2	0.26** (0.04)			
	Q8_3	0.68** (0.07)			
Worries about school resumption	Q1a_1	0.55** (0.03)	0.99	0.03	0.74
	Q1a_3	0.70** (0.03)			
	Q1a_5	0.69** (0.03)			
	Q1a_6	0.46** (0.03)			
	Q1b_1	0.58** (0.03)			
Online learning self-efficacy during the school suspension	Q7_1	0.60** (0.03)	1.00	0.04	0.75
	Q7_2	0.79** (0.02)			
	Q7_3	0.75** (0.03)			
	Q7_4	0.48** (0.03)			
Acquisition of digital skills during the school suspension	Q6_1	0.73** (0.02)	1.00	0.00	0.84
	Q6_2	0.74** (0.02)			
	Q6_3	0.91** (0.02)			

**p < 0.01.

**TABLE 4:** CFA for student outcome variables (secondary schools).

Variables	Items	Factor loadings (SE)	CFI	RMSEA	Reliability (Ω)
Cognitive-emotional regulation during the school suspension	Q9_1	0.45** (0.05)	1.00	0.00	0.71
	Q9_2	0.69** (0.05)			
	Q9_3	0.85** (0.04)			
Worries about school resumption	Q1_1	0.65** (0.03)	0.97	0.06	0.73
	Q1_3	0.75** (0.03)			
	Q1_5	0.62** (0.03)			
	Q1_6	0.49** (0.03)			
	Q1_7	0.51** (0.03)			
	Q1_10	0.50** (0.04)			
Online learning self-efficacy during the school suspension	Q8_1	0.55** (0.03)	0.98	0.07	0.82
	Q8_2	0.74** (0.03)			
	Q8_3	0.52** (0.03)			
	Q8_4	0.81** (0.02)			
	Q8_5	0.79** (0.02)			
	Q8_7	0.57** (0.03)			
Acquisition of digital skills during the school suspension	Q7_1	0.80** (0.02)	0.99	0.04	0.77
	Q7_2	0.62** (0.03)			
	Q7_3	0.88** (0.02)			

**p < 0.01.

### Procedure

The eCitizen Education 360 survey was administered during June–July 2020 when schools resumed briefly after 4 months of suspension. Survey data were collected from school leaders, teachers, students, parents, and the e-learning coordinator from each participating school. All schools in Hong Kong were invited to participate in the survey on a voluntary basis. The schools that came on board made decisions, based on their needs and administrative resources, on the specific grade levels and numbers of classes to participate in the survey. Parents of the participating students were invited to complete the parent survey. For the purposes of this study, only matched parent–child data from primary and secondary schools were analyzed. Various aspects of the study, including project details (e.g., study objectives, elements of research methodology that involved human participants) and data-collection (e.g., data sources, recruitment of study participants, risk assessment, informed consent, data-retention) were reviewed and approved by the research team’s university Human Research Ethics Committee.

### Analytical Strategy

The present study employed the three-step LPA (Vermunt, 2010; Asparouhov and Muthén, 2013; Oberski, 2016), using MPlus, to unravel the relationships among family SES, parental involvement, and student outcomes (Figure 1). In the first step, we fitted a measurement model using the nine parenting indicators. In the second step, we ascertained the measurement errors associated with the latent profiles. In the third step, we used the SES indicators as predictors of latent profile membership and compared levels of the student outcomes among the profiles.

## Results

In the sections that follow, LPA results for the matched parent–child sample for primary schools are presented before those for the secondary schools in this section.

### Primary Student Sample

Different model indicators were used to determine the number of latent profiles (Nylund et al., 2007). First, for the matched parent–child primary school sample, the analysis (Table 5) shows that the information criteria improved when the number of profiles was increased from one to five. However, the likelihood ratio tests indicated that the four-profile solution did not differ significantly from the three-profile solution. The entropy was high for all the profiles (0.74–0.85). The percentage of parents in profiles 1 to 3 in the three-profile solution ranged from 27.96 to 43.01% whereas the percentage for profile 4 in the four-profile solution was only 2.69%. Therefore, a three-profile solution (27.96%, 43.01%, and 29.03% of parents in profiles 1–3, respectively) was used to characterize the parents in the primary student sample.

**TABLE 5:** LPA model fit indicators (primary schools).

	Information criteria			% change			*p* for likelihood ratio tests			Entropy
No. of latent profiles	AIC	BIC	*n*-Adjusted BIC	AIC	BIC	*n*-Adjusted BIC	LMR	Adjusted LMR	Bootstrap	
1	3863.45	3921.52	3864.50	–	–	–	–	–	–	–
2	3698.82	3789.14	3700.46	4.26	3.38	4.24	0.01	0.01	0.00	0.74
3	3595.08	3717.66	3597.30	2.80	1.89	2.79	0.04	0.04	0.00	0.80
4	3542.27	3697.10	3545.07	1.47	0.55	1.45	0.40	0.41	0.00	0.85
5	3501.37	3688.46	3504.76	1.15	0.23	1.14	0.25	0.25	0.00	0.85

Profile 1 parents were named *Low-engagement* parents because they had the lowest mean levels for most of the home involvement variables (except parent–child relationship during school suspension) and school involvement variables during the school suspension (Table 6). In contrast, profile 2 parents had the highest mean levels for most of the home involvement variables (except parental monitoring) and all of the school involvement variables, so they were named *Comprehensive-Support* parent*s*. Profile 3 parents were named *Home-Monitoring-and-Support* parents to reflect their higher levels of monitoring of children’s online activities and relatively high levels of parent–child communication and parental help with children’s homework and lowest levels for most of the school involvement variables.

**TABLE 6:** Descriptives for parenting variables (primary schools).

	M(SE) in latent profiles		
	1. *Low-engagement* parents (*n* = 52; 27.96%)	2. *Comprehensive-Support* parents (*n* = 80; 43.01%)	3. *Home-Monitoring-and-Support* parents (*n* = 54; 29.03%)
**Home involvement**			
Parent–child communication before school suspension	3.04** (0.17)	3.77** (0.07)	3.69** (0.11)
Parental monitoring of children’s online activities before school suspension	2.61** (0.19)	3.84** (0.08)	4.06** (0.20)
Parental monitoring of children’s online activities during school suspension	2.58** (0.17)	3.82** (0.09)	3.84** (0.20)
Parental help with homework during school suspension	2.65** (0.14)	3.38** (0.09)	3.03** (0.12)
Parent–child relationship during school suspension	3.39** (0.09)	3.55** (0.08)	3.27** (0.11)
**School involvement**	2.29** (0.09)	3.02** (0.09)	1.90** (0.10)
Parent–teacher interactions before school suspension	1.80** (0.12)	2.65** (0.11)	1.71** (0.11)
Parental participation in school activities before school suspension	2.38** (0.11)	3.07** (0.08)	1.88** (0.13)
Parent–teacher interactions during school suspension	1.33** (0.06)	1.99** (0.09)	1.33** (0.07)
Parental participation in school activities during school suspension	3.04** (0.17)	3.77** (0.07)	3.69** (0.11)

**p < 0.01.

The three SES indicators (general and home learning resources, parental education) were used to predict parents’ membership in the typology. Results (Table 7) show that none of the SES indicators predicted parents’ membership in the typology at the 0.05 level (with *Comprehensive-Support* parents (profile 2) used as the reference group for comparison). Table 8 presents the levels of student outcomes associated with the four profiles. Pairwise comparisons of profile means of student outcomes (Table 9), conditional on average SES levels, show that students with profile 2 parents had the lowest whereas students with profile 3 parents had the highest mean level of online learning self-efficacy during the school suspension. There were no other significant differences in mean levels of the other three student outcomes (acquisition of digital skills, cognitive-emotional regulation, worries about school resumption) among students associated with the different profiles at the 0.05 level.

**TABLE 7:** SES and latent profile membership (primary schools).

	Logits	Odds ratio
Latent profile 1 (*Low-engagement* parents)		
General home resources	−0.51 (0.32)	0.60
Home learning resources	0.10 (0.33)	1.10
Parental education	−0.03 (0.21)	0.97
Latent profile 3 (*Home-Monitoring-and-Support* parents)		
General home resources	0.35 (0.32)	1.42
Home learning resources	0.50 (0.40)	1.66
Parental education	0.30 (0.19)	1.34

*Reference category is latent profile 2 (Comprehensive-Support parents).*

**TABLE 8:** Descriptives for of student outcomes (primary schools).

	Latent Profiles		
	1 *Low-engagement* parents	2 *Comprehensive-Support* parents	3 *Home-Monitoring-and-Support* parents
		M(SD)	
Cognitive-emotional regulation during the school suspension	−0.14 (0.60)	0.05 (0.75)	0.00 (0.82)
Worries about school resumption	0.09 (0.71)	−0.10 (0.80)	−0.22 (0.66)
Online learning self-efficacy during the school suspension	0.04 (0.68)	−0.07 (0.66)	0.26 (0.52)
Acquisition of digital skills during the school suspension	−0.08 (0.7)	−0.08 (0.69)	0.07 (0.75)

*Means indicated are factor scores of student outcomes.*

**TABLE 9:** Comparison of student outcomes (primary schools).

	Mean difference (SE)
**Cognitive-emotional regulation during the school suspension**	
Profile 1 – Profile 2	−0.19 (0.13)
Profile 1 – Profile 3	−0.13 (0.14)
Profile 2 – Profile 3	0.04 (0.13)
**Worries about school resumption**	
Profile 1 – Profile 2	0.19 (0.13)
Profile 1 – Profile 3	0.31 (0.15)
Profile 2 – Profile 3	0.12 (0.13)
**Online learning self-efficacy during the school suspension**	
Profile 1 – Profile 2	0.12 (0.11)
Profile 1 – Profile 3	−0.21 (0.12)
Profile 2 – Profile 3	−0.33** (0.11)
**Acquisition of digital skills during the school suspension**	
Profile 1 – Profile 2	0.00 (0.13)
Profile 1 – Profile 3	−0.15 (0.14)
Profile 2 – Profile 3	−0.15 (0.13)

*Conditional means (with SES covariates as controls) used in computing differences between latent profiles.*

**p < 0.01.

### Secondary Student Sample

Next, results for the matched parent–child secondary school sample are presented. The analysis (Table 10) shows that the information criteria improved when the number of profiles was increased from one to six. However, the likelihood ratio tests indicated that the five-profile solution did not differ significantly from the four-profile solution. The entropy was high for all the profiles (0.82–0.87). The percentage of parents in profiles 1 to 4 in the four-profile solution ranged from 11.27 to 36.05% whereas the percentage for profile 5 in the five-profile solution was only 2.25% (21 parents). Therefore, a four-profile solution (36.05%, 28.54%, 24.14%, and 11.27% of parents in profiles 1–4, respectively) was used to characterize the parents in the secondary student sample.

**TABLE 10:** LPA model fit indicators (secondary schools).

	Information criteria			% change			*p* for likelihood ratio tests			Entropy
No. of latent profiles	AIC	BIC	*n*-adjusted BIC	AIC	BIC	*n*-adjusted BIC	LMR	Adjusted LMR	Bootstrap	
1.	20533.42	20620.49	20563.33	–	–	–	–	–	–	–
2.	18936.34	19071.78	18982.86	7.78%	7.51%	7.69%	0	0	0	0.87
3.	18193.47	18377.29	18256.61	3.92%	3.64%	3.83%	0	0	0	0.84
4.	17886.97	18119.16	17966.72	1.68%	1.40%	1.59%	0.04	0.03	0	0.82
5.	17629.14	17909.71	17725.50	1.44%	1.16%	1.34%	0.11	0.11	0	0.83
6.	17420.44	17749.38	17533.42	1.18%	0.90%	1.08%	0.11	0.11	0	0.85

Profile 1 parents were named *Low-engagement* parent*s* because they had the lowest mean levels of home and school involvement variables among the four profiles (Table 11). In contrast, profile 4 parents had the highest mean levels of parental home and school involvement variables, so they were named *Comprehensive Support* parents. Profile 2 parents were named *Balanced-Home-and-School-Support* parents because they were, on balance, involved both in school and at home. Profile 3 parents were named *Home-Monitoring-and-Support* parents to reflect their higher levels of home monitoring and support when compared to profile 2 parents.

**TABLE 11:** Descriptives for parenting variables (secondary schools).

	M(SE) in latent profiles			
	1. *Low-engagement* parents (*n* = 336; 36.05%)	2. *Balanced-Home-and-School Support* parents (*n* = 266; 28.54%)	3. *Home-Monitoring-and-Support* parents (*n* = 225; 24.14%)	4. *Comprehensive-support* parents (*n* = 105; 11.27%)
**Home involvement**				
Parent–child communication before school suspension	2.64** (0.07)	3.22** (0.06)	3.57** (0.08)	3.68** (0.09)
Parental monitoring of children’s online activities before school suspension	1.64** (0.04)	2.55** (0.13)	3.34** (0.09)	3.57** (0.11)
Parental monitoring of children’s online activities during school suspension	1.56** (0.04)	2.44** (0.14)	3.21** (0.08)	3.56** (0.13)
Parental help with homework during school suspension	1.95** (0.04)	2.54** (0.07)	2.65** (0.06)	3.09** (0.09)
Parent–child relationship during school suspension	3.12** (0.05)	3.39** (0.05)	3.43** (0.05)	3.55** (0.08)
**School involvement**				
Parent–teacher interactions before school suspension	1.46** (0.05)	2.40** (0.07)	1.60** (0.09)	3.17** (0.12)
Parental participation in school activities before school suspension	1.46** (0.04)	2.19** (0.09)	1.71** (0.08)	3.12** (0.13)
Parent–teacher interactions during school suspension	1.35** (0.04)	2.24** (0.07)	1.53** (0.10)	3.07** (0.13)
Parental participation in school activities during school suspension	1.16** (0.02)	1.68** (0.07)	1.27** (0.05)	2.68** (0.18)

**p < 0.01.

The three SES indicators were used to predict parents’ membership in the typology. Results (Table 12) showed that, when compared to profile 4 (*Comprehensive-Support* parents), parents with more home learning resources were 1.89 times more likely to belong to profile 3, *p* < 0.05. General home resources and parental education did not predict membership in the typology at the 0.05 level. Table 13 presents the levels of student outcomes associated with the four profiles. Pairwise comparisons of profile means of student outcomes (Table 14), conditional on average SES levels, show that students with profile 2 parents had the lowest mean levels for cognitive-emotional regulation, online learning self-efficacy, and acquisition of digital skills, and the highest mean level of worries about school resumption. Students with profiles 3 and 4 parents had the highest mean levels for cognitive-emotional regulation, online learning self-efficacy, and acquisition of digital skills. Additionally, students with profile 3 parents had the lowest mean level of worries about school resumption.

**TABLE 12:** SES and latent profile membership (secondary schools).

	Logits	Odds ratio
Latent profile 1 (*Low-engagement* parents)		
General home resources	−0.16 (0.22)	0.85
Home learning resources	−0.10 (0.25)	0.91
Parental education	−0.13 (0.12)	0.88
Latent profile 2 (*Balanced Home-and-School-Support* parents)		
General home resources	−0.22 (0.24)	0.80
Home learning resources	−0.46 (0.27)	0.63
Parental education	−0.14 (0.14)	0.87
Latent profile 3 (*Home-Monitoring-and-Support* parents)		
General home resources	0.17 (0.24)	1.18
Home learning resources	0.64* (0.28)	1.89
Parental education	−0.17 (0.14)	0.84

*Reference category is latent profile 4 (Comprehensive-support parents).*

*p < 0.05.

**TABLE 13:** Descriptives for of student outcomes (secondary schools).

	Latent profiles			
	1. *Low-engagement* parents	2. *Balanced-Home-and-School-Support* parents	3. *Home-Monitoring-and-Support* parents	4. *Comprehensive-Support* parents
	M(SD)			
Cognitive-emotional regulation during the school suspension	−0.03 (0.48)	0.00 (0.46)	0.04 (0.5)	0.01 (0.52)
Worries about school resumption	0.01 (0.53)	−0.01 (0.51)	−0.08 (0.56)	−0.04 (0.51)
Online learning self-efficacy during the school suspension	−0.02 (0.52)	0.08 (0.47)	0.09 (0.52)	0.18 (0.49)
Acquisition of digital skills during the school suspension	−0.04 (0.58)	0.09 (0.56)	0.09 (0.64)	0.12 (0.62)

*Means indicated are factor scores of student outcomes.*

**TABLE 14:** Comparison of student outcomes (secondary schools).

	Mean difference (SE)
**Cognitive-emotional regulation during the school suspension**	
Profile 1 – Profile 2	0.38** (0.08)
Profile 1 – Profile 3	−0.24** (0.07)
Profile 1 – Profile 4	−0.09 (0.08)
Profile 2 – Profile 3	−0.62** (0.06)
Profile 2 – Profile 4	−0.47** (0.09)
Profile 3 – Profile 4	0.15 (0.09)
**Worries about school resumption**	
Profile 1 – Profile 2	−0.41** (0.08)
Profile 1 – Profile 3	0.27** (0.09)
Profile 1 – Profile 4	0.07 (0.08)
Profile 2 – Profile 3	0.68** (0.06)
Profile 2 – Profile 4	0.49** (0.08)
Profile 3 – Profile 4	−0.19* (0.09)
**Online learning self-efficacy during the school suspension**	
Profile 1 – Profile 2	0.46** (0.09)
Profile 1 – Profile 3	−0.34** (0.09)
Profile 1 – Profile 4	−0.24** (0.08)
Profile 2 – Profile 3	−0.79** (0.06)
Profile 2 – Profile 4	−0.70** (0.09)
Profile 3 – Profile 4	0.10 (0.09)
**Acquisition of digital skills during the school suspension**	
Profile 1 – Profile 2	0.45** (0.12)
Profile 1 – Profile 3	−0.36** (0.08)
Profile 1 – Profile 4	−0.23** (0.09)
Profile 2 – Profile 3	−0.81** (0.09)
Profile 2 – Profile 4	−0.68** (0.12)
Profile 3 – Profile 4	0.13 (0.10)

*Conditional means (with SES covariates as controls) used in computing differences between latent profiles.*

*p < 0.05; **p < 0.01.

## Discussion

Results from the present study are discussed in this section. Specifically, the discussion will focus on three aspects, namely nuanced patterns of parental involvement for both the matched parent–child primary and secondary school samples, parental monitoring and support in homes with more learning resources, and plausible benefits of parental home monitoring and support for students’ socioemotional well-being and online learning during the school suspension in Hong Kong.

First, the present study finds that there were typologies of parental involvement during the pandemic-induced school suspension when teaching and learning was taken online in Hong Kong. The typologies comprised three categories of parents for the matched parent–child primary school sample (*Low-engagement*, *Comprehensive-Support*, *Home-Monitoring-and-Support*) and four categories of parents for the matched parent–child secondary school sample (*Low-engagement*, *Balanced-Home-and-School Support*, *Home-Monitoring-and-Support*, *Comprehensive-Support*). The typologies indicate that parents are not necessarily highly involved or totally uninvolved in their children’s learning. Instead, they can have different combinations of home and school involvement practices, possibly to address their children’s learning needs. These results add to an emerging body of studies illustrating the value of using latent class/profile analysis to unravel the complexity in parenting during the pandemic. For example, Pratama and Firmansyah’s (2021) identified a typology of parents varying in their attitudes toward students’ home learning (*Disengaged*, *Positive*, *Negative*) in Indonesia. Martin-Storey et al. (2021) found that there were four latent profiles of Canadian adolescents varying in their perceptions of change in family relationship quality (*Low Change*, *Improvement Only*, *Moderate Instability*, *High Instability*).

Second, the present study shows that, among the three SES indicators examined, only the indicator “home learning resources” was positively associated with membership in the parenting typology for the secondary student sample. Specifically, parents whose secondary school children reported having more learning (not general) resources were more likely to be monitoring and supporting their children’s online learning. In contrast, non-learning-related SES indicators (e.g., general home resources, parental education) were not predictive of membership in the typology for both primary and secondary student samples. Home general resources may be indicative of the economic aspects of family SES whereas home learning resources reflect parents’ priority given to learning resources. The former is less amenable to school interventions whereas schools can provide parental education programs aimed at supporting families’ effective use of home educational resources for student learning.

The results suggest that parents who are involved at home may procure more learning resources to address their older children’s learning needs. The availability of home learning resources also enables parents to plan and structure effective learning activities for their children (Chen C. Y. C. et al., 2022). More fundamentally, the availability of home learning resources may indicate that parents have more positive attitudes toward students’ home-based online learning (Pratama and Firmansyah, 2021), and therefore, these parents may have higher levels of monitoring of and support for their children’s online learning.

Another set of results from the present study shows that, compared to peers associated with other parental latent profiles, primary school students of *Home-Monitoring-and-Support* parents had the highest levels of online learning self-efficacy during the school suspension. These results suggest that parental monitoring and support serves a protective function for students’ online learning during the pandemic. This finding adds to the nuanced findings in the literature on associations between parental monitoring and student learning (Patall et al., 2008; Hill and Tyson, 2009). For example, Patall et al. (2008) found that parental homework help was positively associated with the achievement of elementary and high school students but not for middle school students’ achievement. They also reported that parental homework help was positively associated with students’ verbal achievement but negatively associated with students’ mathematics achievement.

Parental monitoring and support is particularly important for primary school students’ online learning during the pandemic-induced school suspension. This is because these younger students may have difficulties completing distance learning tasks at home due to lack of learning interests and limitations of home learning environments (Lau and Lee, 2021). Consequently, parents can play an important role by being teachers, autonomy-supportive coaches, and interveners (Knopik et al., 2021). Spear et al. (2021) provided additional insights from teachers’ perspectives on what parents need to do to monitor and support primary school students’ learning during the pandemic-induced school suspension. Specifically, teachers expected parents to enable students’ access to learning resources provided by teachers and participate in or supervise the completion of learning activities assigned by teachers. When parents monitor and support their children’s learning, they also benefit from this commitment. This is evident in Akinrinmade et al.’s (2021) study which found that parents of primary school students in Nigeria acknowledged that the extra time they spent together with their children during the pandemic-induced school suspension enabled them to improve the parent–child relationship and spend time discussing moral development and discipline with their children.

The benefits of parental home monitoring and support are even greater for secondary school students. Specifically, our results show that, compared to peers associated with other latent profiles of parents, secondary school students of *Home-Monitoring-and-Support* parents had the highest levels of acquisition of digital skills, had the highest levels of cognitive-emotional regulation and self-efficacy in online learning, and were the least worried about school resumption during the school suspension. Parents who commit time and resources to monitor and support their children’s learning are better able to understand their children’s learning needs. For example, Demir and Demir’s (2021) study found that elementary and secondary school students in Turkey had lower levels of motivation (because of Internet connection problems and social isolation) during the pandemic-induced school suspension, so parents played an important role by ensuring that their children followed the online lessons and motivating their children. In the process of monitoring and providing support, parents had a better understanding of their children’s education and appreciated the value of their children’s schooling. Tarsuslu et al. (2021) reported that almost half of the parents in their sample in Turkey indicated that spending time at home with their children during the pandemic-induced school suspensions strengthened their relationships with their children and the majority of parents perceived that the time spent together at home with their children enabled them to take better care of their children.

In contrast to these results alluding to the plausible benefits of parental involvement (particularly home monitoring and support), our analysis shows that primary students from the three parental latent profiles (*Low-engagement*, *Comprehensive-Support*, *Home-Monitoring-and-Support* parents) did not differ in their perceived acquisition of digital skills and cognitive-emotional regulation during the school suspension and worries about school resumption. There are no apparent reasons for this pattern of findings. However, the different patterns of results between the primary and secondary school samples underscore the importance for research on familial influences (SES, parental involvement) and student outcomes to consider contextual factors (e.g., students’ grade levels or age) (Tan et al., 2019). Previous studies suggest that, compared to secondary school students, primary school students may be more dependent on parental interventions and therefore, parents need to work harder to understand their children’s developmental and learning needs, cultivate a positive parent–child relationship, and be more proactive in their parenting (Knopik et al., 2021; Lau and Lee, 2021; Spear et al., 2021). Future research can expand the scope of investigation of parenting influences beyond parental involvement to identify aspects of parenting that can enhance the online learning of primary students during school suspension.

## Conclusion

The present study unravels complex relationships among SES, parental involvement, and learning outcomes for 186 primary and 932 secondary school students and their parents during the prolonged period of COVID-19-related school suspension in Hong Kong. The three-step LPA found different patterns of associations among these variables for the primary and secondary school samples. For the primary school sample, students’ SES did not predict membership in the parental involvement typology, but students whose parents provided more home monitoring and support had the highest level of online self-efficacy. As for the secondary student sample, students whose parents provided more home monitoring and support tended to have access to more home learning resources and they had the highest levels of online self-efficacy, acquisition of digital skills, and cognitive-emotional regulation, and were the least worried about school resumption.

These results contribute to theory and practice in three ways. First, it underscores the importance of elucidating nuanced patterns of parental involvement for students from different grade levels (Tan, 2018; Martin-Storey et al., 2021; Pratama and Firmansyah, 2021). Indeed, the results show that the typology of parental involvement practices differed between the matched parent–child primary and secondary school samples, and there were different levels of student outcomes among various parent latent profiles and between the primary and secondary school samples. The second contribution is the identification of *Home-Monitoring-and-Support* parents as the most plausible latent profile of parents that provides a protective function for students’ home-based online learning during the pandemic-induced school suspension. Secondary school students associated with this latent profile had the highest levels of online learning (online learning self-efficacy, acquisition of digital skills) and socioemotional well-being (cognitive-emotional regulation, least worries about school resumption) while primary school students associated with this latent profile had the highest level of online learning self-efficacy. These results are important because they indicate that some aspects of parental involvement (e.g., home monitoring and support) may be more beneficial than others for student learning during the challenging times of school suspension. Therefore, parents can consider how they can devote more of their limited energy, time, and material resources to provide quality monitoring and support as opposed to other parental involvement activities. Lastly, the present study points the way forward for schools to support parents during future school suspensions. School psychologists and mental health professionals can develop intervention programs to enable parents to effectively monitor and support students’ home online learning (Anderson et al., 2021; Hatzichristou et al., 2021). These programs are critical to optimize student well-being and online learning during future school suspensions due to pandemics (including COVID-19) or other compelling reasons. They contribute to the development of resilient education systems that are more fit-for-purpose in future contingencies.

Results from the present study have to be read with three limitations in mind. The first limitation is that the data was cross-sectional, so the results should not be taken to be evidence of causal relationships. Second, only three indicators (parental education, home general and educational resources) were used to measure SES, so there may be other patterns of SES-parental involvement associations if parental occupational status and family income were included as SES indicators. Third, the present study focused only on specific aspects of students’ online learning (self-efficacy, acquisition of digital skills) and socioemotional well-being (cognitive-emotional regulation during school suspension, worries about school resumption). There are other aspects of student outcomes that are not investigated in the present study.

Moving forward, researchers can include more SES indicators (beyond parental education and home resources) to provide a comprehensive examination of the association between SES and parental involvement in students’ home-based online learning during the pandemic-induced school suspension. There is also a need for research to investigate how different parenting styles can moderate the influence of parental involvement in supporting students’ online learning. Furthermore, researchers can examine other student outcomes such as academic achievement and long-term socioemotional well-being arising from the school suspension. Lastly, future studies can employ qualitative studies to compare perspectives of different stakeholders (parents, students, teachers) with regards to how and why parental involvement may contribute to students’ well-being and online learning outcomes. These studies can also collect longitudinal data to obtain insights on how these perspectives may change over different waves of school suspensions.

## Data Availability Statement

The original contributions presented in this study are included in the article/supplementary material, further inquiries can be directed to the corresponding author/s.

## Ethics Statement

The studies involving human participants were reviewed and approved by The University of Hong Kong Human Research Ethics Committee. Written informed consent to participate in this study was provided by the participants’ legal guardian/next of kin.

## Author Contributions

All authors listed have made a substantial, direct, and intellectual contribution to the work, and approved it for publication.

## Conflict of Interest

The authors declare that the research was conducted in the absence of any commercial or financial relationships that could be construed as a potential conflict of interest.

## Publisher’s Note

All claims expressed in this article are solely those of the authors and do not necessarily represent those of their affiliated organizations, or those of the publisher, the editors and the reviewers. Any product that may be evaluated in this article, or claim that may be made by its manufacturer, is not guaranteed or endorsed by the publisher.

## Footnotes

1 Respondents to the parent survey could be parents or guardians. A large majority of the responses came from parents (98.92% in primary school sample and 96.57% in secondary school sample).
